# Emotional Adjustment among Adolescent Students with and without Specific Learning Disabilities

**DOI:** 10.3390/children10121911

**Published:** 2023-12-11

**Authors:** Isaías Martín-Ruiz, María-José González-Valenzuela, Lidia Infante-Cañete

**Affiliations:** Department of Developmental and Educational Psychology, Faculty of Psychology and Speech Therapy, University of Málaga, 29016 Málaga, Spain; ismar@uma.es (I.M.-R.); valenzu@uma.es (M.-J.G.-V.)

**Keywords:** internalising problems, externalising problems, personal resources, emotional adjustment, specific learning disabilities, adolescence

## Abstract

Adolescence is a psychologically vulnerable stage in which problems of emotional adjustment and psychological well-being can appear. The aim of this study is to analyse the relationship and comparison of emotional deficits and resources among adolescents with or without specific learning disabilities. We evaluated 80 students distributed into two groups: 40 adolescents with specific learning disabilities and 40 normative adolescents matched with the previous group in terms of age, sex, and school year. The study variables are internalising problems (anxiety and depression), externalising problems (aggression, anger control, defiant behaviour, and antisocial behaviour), and personal resources (self-esteem, social competence and integration, and awareness of problems), evaluated by means of the SENA test. The results indicate a positive relationship between externalising and internalising problems and a negative relationship between the latter and some personal resources in both groups. We also found that adolescents with specific learning disabilities displayed more internalising and externalising symptoms than their peers, greater awareness of their emotional difficulties, and lower self-esteem and social integration and competence. The findings highlight the need for preventive interventions that promote the psychological well-being and mental health of adolescents within the school setting at an early age.

## 1. Introduction

Adolescence is a period of bio-psycho-social change in which personality development occurs [1]. This process of identity construction influences psychological well-being, emotional adjustment, the way one deals with everyday situations, and the use of different personal resources. In a broad sense, adolescence is a time of vulnerability, which one may be more susceptible to the influence of poor personal resources and emotional difficulties. Personal resources are essential for the development of psychological well-being, as they act as a source of protection against age-specific changes or the appearance of stressors. Some of these scarcities in personal resources that occur in adolescence are low self-esteem or positive self-perception and poor social competence and skills, preventing the subject from responding to social demands in an adequate way [1]. Regarding emotional difficulties, some authors have established two categories [2,3]. On the one hand, there are internalising problems, including symptoms such as low mood, worry, and withdrawal, which overlap with each other and are shared symptoms in anxiety and depression [2,3,4]. On the other hand, there are externalising emotional deficits, which refer to aggressive behaviours, including breaking established social rules and behavioural or defiant disorders [2,3]. Emotional problems have a high rate of occurrence in adolescence, with between 13 and 16% of teenagers presenting anxiety, depression, or behavioural disorders [1,2,3,4,5,6]. In general, it is common for an adolescent to present a combination of internalising and externalising symptomatology and not just one type of emotional problem, especially if prevention or early intervention measures have not been implemented [2,3,4]. Adolescents tend to present emotional disorders, particularly those of the anxious-depressive type [1,2,3,4,5], but half of them are not usually correctly identified and go unnoticed. As this is a time of transition and change, social relations and other personal traits, such as self-conception, are fluctuating and volatile [4,5], and this can negatively affect self-image. In contrast, self-esteem and social skills can play a protective and relevant role in the development of emotional stability and psychological well-being among adolescents [7,8] since they can receive positive self-messages from themselves and attain a social support network to help them through emotionally complex situations. In addition, adolescents who have a good although not extensive social network are more likely to have good self-esteem, good social skills, and fewer emotional problems, while those who do not have one tend to have lower self-esteem, fewer social skills, and more emotional imbalances [7,8,9,10]. In short, there is a strong relationship between personal resources and emotional problems, while, particularly in adolescence, there is a negative relationship between them and a positive relationship between the types of emotional problems (internalising and externalising) [8,9,10]. 

These emotional characteristics may be aggravated in certain more-vulnerable groups, such as people with specific learning disabilities (SLD) [11,12,13]. Specific Learning disabilities (SLD) refer to a neurodevelopmental disorder that begins in childhood, characterised by a deficit in reading (regarding accuracy, fluency, or reading comprehension), writing (with respect to spelling or writing), or mathematics (concerning number sense, calculation, or mathematical reasoning). Therefore, this term refers to specific, significant, and persistent difficulties in learning academic skills corresponding to performance significantly below the expectations for one’s age and overall intellectual level. SLD have been defined as significant disorders in the cognitive processes involved in learning that substantially interfere with school performance and in daily and school activities and are not determined by an intellectual, physical, or sensory disability; severe emotional disorder; lack of opportunities; or sociocultural factors [14]. The cognitive-linguistic manifestations of students with SLD can relate to visual-spatial perception, auditory perception, or speech perception; verbal or phonological memory; knowledge of letters; prosody; phonological awareness; rapid automatic naming; executive function; and vocabulary, among others [15,16]. These academic and cognitive-linguistic deficits can produce a relevant and limiting emotional effect, which can trigger school drop-out, mental health difficulties, and high levels of psychological distress [11,12,13]. Therefore, some studies consider that students with SLD present deficits in social competence, behavioural disorders, anxiety or depression, and scarce personal resources, produced through the repeated experience of school failure that these deficits precipitate [11,12,13,17,18,19]. Research indicates that exposure to repeated experiences of low school performance and continued failure can be a trigger for emotional difficulties to appear [20,21]. Likewise, theories of attributions in the face of repeated failure create a maladaptive thinking schema with an external and unstable locus of control for success and an internal and stable locus of control for failure [11,12,13,20,21,22]; hence, students with specific learning disabilities may present these clinical manifestations more frequently than students without SLD.

Regarding internalising problems, the study by Zuppardo et al. [23] revealed that teenagers with SLD have higher scores in anxiety and depression, withdrawal, and somatic complaints than their peers. Donolato et al. [24] carried out a meta-analysis on emotional adjustment among students with and without SLD, finding disparate results. They showed that some studies have found that adolescents with SLD have higher scores in internalising problems than the control subjects, with a 60% higher probability [24,25]. However, other studies did not find different levels of anxiety or depression among adolescents with and without SLD [11,12], probably because the authors used measures linked to mood and more stable and general personality traits that do not indicate differences in internalising emotional symptoms between these groups. Another of the studies analysed [25] found no conclusive results among adolescents with and without SLD in terms of anxiety and depression through self-reporting measures, finding different results according to the test used. They found no differences with general personality tests; however, with more specific tests, they did find differences between the groups.

Regarding externalising problems, several studies indicate the high prevalence of externalising emotional difficulties among students with SLD [20,21,26], finding up to three times the rate of occurrence among samples of adolescents with SLD [20]. Alemany [18] highlighted the presence of behavioural disorders (21%), frustration, anger (4.6%), and conflicts with the law (1.5%) among adolescents with SLD, while Zuppardo et al. [23] pointed out that adolescents with SLD score significantly higher than their peers in aggressive behaviour, although it was not significant in terms of criminal behaviour. In the same vein, the study by Donolato et al. [24] revealed that most of the studies analysed in their meta-analysis highlight externalising emotional difficulties in adolescents with SLD, who are up to 61.7% more likely to present behavioural disorders than their peers.

On the other hand, regarding personal resources, some studies have found that students between twelve and sixteen years old with SLD have negative self-esteem at the academic level [27,28] and as a general construct [21,23]. However, other studies did not find any differences in general self-concept, or at a specific level, such as family or emotional, in seventeen-year-old students with SLD compared to their peer group [11]. Regarding competence and social skills, some studies found that children with SLD tend to show more social difficulties than their peers and receive more negative nominations than their peers, presenting low-level social skills that do not help them integrate with their peers [20,28,29].

In short, the existing research indicates that the existence of emotional imbalances and scarce personal resources are some of the traits of adolescents with SLD, although the results differ greatly depending on age and the way emotional adjustment and psychological well-being were measured. There are also studies conducted with normative students that show the existence of these emotional imbalances and this shortage of personal resources, although such studies are less frequent. On the other hand, discrepancies have been found regarding the differences found in some variables used to evaluate emotional adjustment in adolescence between students with and without SLD. It should also be noted that most studies analyse socioemotional deficits regardless of personal resources, with the latter being less studied.

For these reasons, the aim of this study is to analyse the relationship and comparison of certain emotional problems (internalising and externalising) and personal resources among students with and without SLD. A positive relationship was expected to exist between internalising and externalising problems, and a negative one was posited to exist between such problems and the personal resources studied regarding students with and without SLD. Adolescents with SLD were also expected to have more internalising and externalising problems and fewer personal resources than their peers without SLD.

## 2. Materials and Methods

### 2.1. Participants

The study population comprised students in compulsory secondary education in state-funded and state-subsidised private schools from average sociocultural areas in Malaga. The schools that participated in the research were selected by means of stratified sampling by catchment areas in the province of Malaga according to the Junta de Andalucia [30], randomly selecting four schools that agreed to participate voluntarily, comprising three from the city of Malaga and one from the wider province. 

From these schools, the sample of participants was selected, finally comprising two groups of students: one with specific learning disabilities (SLD Group) and another consisting of students that did not present specific learning difficulties (NSLD Group). 

The participants in the SLD group presented specific learning difficulties in literacy or mathematics according to the psycho-pedagogical assessment reports carried out by school counsellors based on the inclusion criteria of Andalusia’s Regional Department for Education [31]. The participants of the NSLD Group did not present any type of specific educational support needs. They were of the same age and gender as the other group and were selected at random.

The study sample was composed, therefore, of 80 students distributed into two groups. The SLD Group was made up of 40 adolescents with SLD, of whom 32 were male and 8 were female, with an average age of 14.3 years (*SD* = 1.16). The NSLD Group was made up of 40 adolescents who did not present SLD, were of the same age (*M* = 14.40 and *SD* = 1.3) and were also matched with the SLD group in terms of school year and sex. There were no statistically significant differences between both groups with regard to age [*t*(78) = −0.14 and *p* = 0.879)] or sex [*λ^2^*(78) = 0.00 and *p* = 1)].

### 2.2. Instruments

The study variables were evaluated using the Internalising Problems, Externalising Problems, and Personal Resources scales of the Evaluation System for Children and Adolescents (SENA) questionnaire developed by Fernández-Pinto et al. [32]. The test uses Likert-type items with a score that ranges from 1 (never or almost never happens) to 5 (always or almost always happens) points for each item. Table 1 presents the quantities of each scale’s items, the lowest and highest assessments for each factor, and the scales and their theoretical dependability. Additionally, it displays the reliability of the test obtained in the sample of the study. Adequate and similar levels of the reliability indices of the test and the results obtained in the study were observed.

Internalising problems (INTER) refer to the presence of symptoms associated with anxiety and depression. The variable Anxiety (ANX) refers to manifestations of generalised subjective discomfort, persistent and recurrent worries, and emotional tension, including behaviours such as fear or panic. The variable depression (DEP) indicates the presence of symptoms such as dysphoria, anhedonia, anergia, feelings of uselessness and guilt, helplessness, and thoughts related to death and suicide. 

Externalising (EXTER) problems refer to aggressive behaviours, anger control, defiant behaviour, and antisocial behaviour. The variable aggression (AGR) refers to the presence of behaviours that denote low empathy and can manifest in a certain cruelty and interpersonal aggression towards others. The variable Anger Control (ANG) indicates the exaggerated and inappropriate experiencing of aggressive-impulsive behaviours, feelings of anger, and perceived loss of control. The variable Defiant Behaviour (DEF) refers to behaviours of defiance and opposition to authority figures, including milder behaviours of disobedience. Finally, the variable Antisocial Behaviour (ANT) refers to the presence of a pattern of behaviours that violate the basic rights of other people and the basic norms of coexistence.

Finally, Personal Resources (PR) refer to adolescents’ abilities to resolve and cope with emotional difficulties they encounter. Three variables have been considered. The variable Self-esteem (SEL), which indicates a person’s degree of satisfaction with themselves, their valuation, and personal adjustment. The variable Social Integration and Competence (SOC) refers to the ability to relate effectively to others, integrate into peer groups, and thus obtain support and reinforcement. Finally, the variable Awareness of Problems (AWE) indicates the degree to which a person can perceive emotional difficulties in their daily lives and be aware of what is happening and whether they should seek help. 

### 2.3. Procedure

After contacting the participating schools, we requested informed consent from the management teams of the schools, as well as from legal guardians from students. 

This research complies with the ethical and deontological code of the University of Malaga Ethics Committee (CEUMA).

The tests were administered during school hours through an online questionnaire that each student completed individually in a 45 min session under the supervision of two Psychology graduates. The instructions and information on the confidentiality of the responses were previously provided. The research team answered any questions that the students might have had. 

### 2.4. Statistical Design and Analysis

The design of this study is observational, descriptive, and comparative, with a cross-sectional cohort and a single data collection. It consists of two groups of participants (with and without SLD) and three study variables (internalising problems, externalising problems, and personal resources). The variables were recoded to avoid differences between the number of items in the different scales and to compare the results in the same range of scores. To do this, the average score for each variable was used, ranging from 1 to 5 points.

First, we performed an analysis of the relationships between variables, using Spearman’s non-parametric rho correlations in both groups because no homogeneity was shown between the variances. Subsequently, we carried out descriptive analyses and determined differences between groups using *t* Welch adjustment test [33] for the same reasons. The effect size of the differences between groups was also calculated using Cohen’s d [34], considering it to be either small (0.20), medium (0.50), or large (greater than 0.80).

Statistical analyses were conducted using the Statistical Package for Social Sciences (SPSS) version 28 [35], and the statistical programme G*Power 3.1 [36] was used to calculate the effect size of the differences. 

## 3. Results

Table 2 presents the correlations and significance indices between the variables of the SLD and NSLD groups in the upper and lower matrix, respectively.

In the SLD group, a high correlation was found between most of the INTER, EXTER, and PR variables. Regarding the INTER variables, a high positive correlation was found between ANX and DEP (*rho* = 0.82 and *p* < 0.001) as well as for all the EXTER variables [e.g., AGR and ANG (rho = 0.83 and *p* < 0.001), ANG and DEF (*rho* = 0.82 and *p* < 0.001), and DEF and AGR (*rho* = 0.67 and *p* < 0.001)], with values between high and medium. As for the variable PR, it was found that all the values had significant correlations, but two of them were positive, SOC and SEL (*rho* = 0.68 and *p* < 0.001), and the other was negative, corresponding to AWE with SEL (*rho* = −0.78 and *p* < 0.001) and with SOC (*rho* = −0.44 and *p* < 0.001). A positive and significant relationship was also observed between all the INTER and EXTER variables (DEP and ANG (*rho* = 0.79 and *p* < 0.001) and ANX and ANT (*rho* = 0.45 and *p* < 0.001), among others), with values between medium and high. A high-level and significant negative relationship was also found between internalising problems and some personal resources, specifically with self-esteem (ANX and SEL (*rho* = −0.79 and *p* < 0.001)) and social competence and integration (DEP and SOC (*rho* = −0.46 and *p* < 0.001), among others), and a positive relationship was found with awareness of problems [ANX (*rho* = 0.77 and *p* < 0.001) and DEP and SOC (*rho* = 0.77 and *p* < 0.001)]. The relationship between the EXTER and PR variables showed more moderate and significantly negative indices among all the variables with respect to self-esteem (e.g., AGR and SEL (*rho* = −0.51 and *p* < 0.001) and DEF and SEL (*rho* = −0.63 and *p* < 0.001), among others) and social competence and integration and significantly with respect to ANT (*rho* = −0.21 and *p* < 0.001) and not significantly with respect to the rest of the variables (e.g., AGR and SOC (*rho* = −0.21 and *p* > 0.05), among others). The variable AWE positively correlated with all the EXTER variables (ANG (*rho* = 0.75 and *p* < 0.001) and ANT (*rho* = 0.60 and *p* < 0.001), among others). 

In the NSLD group, a significant correlation was observed between the INTER variables (ANX and DEP (*rho* = 0.71 and *p* < 0.001)), as well as between all the EXTER variables (ANG and AGR (*rho* = 0.43 and *p* < 0.001), ANG and DEF (*rho* = 0.34 and *p* < 0.05) and ANT and DEF (*rho* = 0.59 and *p* < 0.001), among others), with the majority being mean values. The PR variables showed a positive correlation between SOC and SEL (*rho* = 0.62 and *p* < 0.001) and a non-significant correlation between AWE and SOC (*rho* = −0.30 and *p* > 0.05). Likewise, a significant relationship was observed between some INTER and EXTER variables, such as anger control [(ANG and DEP (*rho* = 0.51 and *p* < 0.001)), ANG and ANX (*rho* = 0.56 and *p* < 0.001), and aggression (AGR and DEP (*rho* = 0.35 and *p* < 0.05)], but not a significant one with defiant behaviour (ANX and DEF (*rho* = 0.30 and *p* > 0.001) and DEP and DEF (*rho* = 0.30 and *p* > 0.001)) or antisocial behaviour (ANX and ANT (*rho* = 0.16 and *p* > 0.001) and ANX and DEF (*rho* = 0.20 and *p* > 0.001)). A significant relationship was also observed between internalising problems and personal resources; specifically, a negative relationship was observed between depression and self-esteem (DEP and SEL (*rho* = −0.61 and *p* < 0.001)) and anxiety and social competence and integration (ANX and SOC (*rho* = −0.40 and *p* < 0.001)) and a positive one was observed with AWE (ANX and AWE (*rho* = 0.53 and *p* < 0.001) and DEP and AWE (*rho* = 0.66 and *p* < 0.001)). The relationship between the EXTER and PR variables presented significant and negative correlations between self-esteem and aggression (AGR and SEL (*rho* = −0.36 and *p* < 0.001)) and defiant behaviour (DEF and SEL (*rho* = −0.52 and *p* < 0.001)) and non-significant ones between anger (ANG and SEL (*rho* = −0.25 and *p* > 0.001)) and antisocial behaviour (ANT and SEL (*rho* = −0.30 and *p* > 0.001)). On the other hand, all the EXTER variables have non-significant relationships with Social Competence and Integration (e.g., AGR and SOC (*rho* = −0.14 and *p* > 0.05) and ANT and SOC (*rho* = −0.50 and *p* > 0.001), among others). The AWE variable only correlated significantly with ANG (*rho* = 0.39 and *p* < 0.05) and did not correlate significantly with AGR (*rho* = 0.24 and *p* < 0.05) or ANT (rho = 0.05 and *p* < 0.05), among others.

The analysis of the descriptive statistics and the comparison of the means between groups can be seen in Table 3. 

The differences between the groups were statistically significant for all the study variables (Table 3).

Regarding the INTER variables, statistically significant differences were found between groups regarding ANX, with a large effect size, and with respect to DEP, with a medium effect size. The scores were higher for all variables in the SLD group (see Table 3).

**Figure 1:**
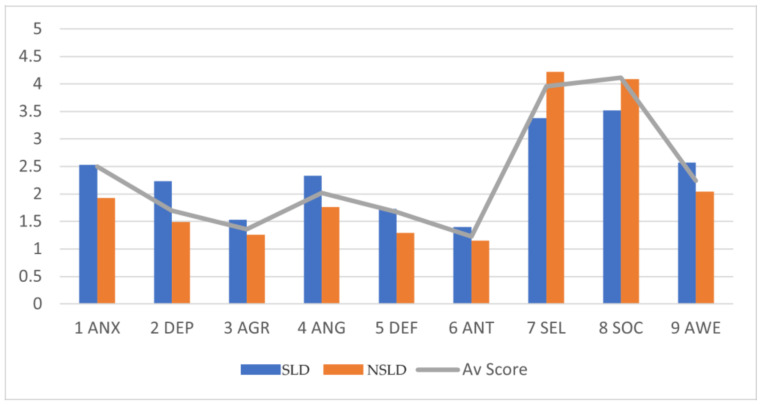
Average scores of the groups for the different variables. Note: SLD Group (specific learning disabilities) and NSLD Group (no specific learning disabilities). Av Score (Average Score), 1 ANX (Anxiety), 2 DEP (Depression), 3 AGR (Aggression), 4 ANG (Anger Control), 5 DEF (Defiant Behaviour), 6 ANT (Antisocial Behaviour), 7 SEL (Self-Esteem), 8 SOC (Social Competence and Integration), and 9 AWE (Awareness of Problems).

Regarding the EXTER variables, statistically significant differences were also observed in terms of AGR, with a medium effect size; regarding ANG, with a large effect size; concerning DEF, with a large effect size; and with regard to ANT, with a medium effect size. The scores were higher for all variables in the SLD group (see Table 3). The SLD Group exhibited higher scores than the NSLD Group regarding the INTER (ANS and DEP) and EXTER (AGR, ANT, DEF, and ANT) variables, as depicted in Figure 1. It is evident that all the scores of the SLD Group are above the expected mean in terms of the INTER and EXTER variables compared to the NSLD group, whose scores were below the expected level. 

Finally, statistically significant differences were also found between the groups in terms of the PR variables, specifically with respect to SEL, with a large effect size; regarding SOC, with a medium effect size; and with respect to AWE, with a medium effect size (see Table 3). The scores were lower or similar for all the variables in the SLD group, except for AWE, which presented an opposite pattern. Thus, for the PR variables, the students of the NSLD Group presented higher scores than the adolescents of the SLD Group in of SEL and SOC, and the opposite was true for AWE (see Figure 1). The scores of the SLD group for the PR variables are below expectations compared to those of the NSLD group, which exceed expectations.

## 4. Discussion

The objective of this study was to analyse the relationship between certain emotional problems (internalising and externalising) and personal resources and compare them among adolescents with and without specific learning disabilities. 

Firstly, we expected to find a positive relationship between internalising and externalising problems and a negative relationship with personal resources in both groups. 

The results show a high positive correlation between internalising (ANX and DEP) and externalising problems (AGR, ANG, DEF, and ANT) in both groups (SLD and NSLD), but with higher indices in the group with SLD. This study reveals that adolescent express anxiety and depression problems, in general, as well as behavioural problems of various kinds, and not just one type in isolation [4,5,6,7]. This pattern of diverse and global symptomatology is stronger in the case of students with SLD, as some other studies have pointed out [23,24]. This study also reveals the positive relationship between SEL and SOC. There is a significant relationship between the variables of self-esteem and socialisation since they are part of the resources with which adolescents can face the new challenges and difficulties of this developmental stage [37,38], and this occurs in a similar way in both groups. However, it does not occur with the AWE variable, which presented a negative relationship with the other personal resources, being significant with respect to SOC only in the SLD group. Adolescents with SLD exhibit a stronger relationship between their awareness of their problems and lower social competence than their peers, so they have a more maladaptive pattern induced by perceiving their emotional difficulties and social problems [22,23,24,25,26,27,28]. Likewise, a high correlation was found between internalising and externalising problems among adolescents with SLD, more so than among their peers. On the other hand, adolescents without specific learning disabilities in some cases presented this relationship with respect to ANG or AGR. Some studies have found that adolescents tend to have internalising and externalising emotional difficulties and rarely present a single type [2,3,4,5]. This relationship between emotional and behavioural problems has greater relevance in the SLD group, where adolescents present more complex and generalised problems than their peers [13,17,18,19,20]. On the other hand, negative relationships were found between internalising emotional difficulties and personal resources and between self-esteem and socialisation, and a positive relationship was found with awareness of their problems, with a higher index in the SLD group. Adolescents who manifest anxiety or depression have lower self-esteem and social competence [4.37] and are aware of their emotions. This relationship is higher among adolescents with SLD due to their greater problems [17,18,19,20,25]. Likewise, externalising problems and personal resources presented a stronger relationship in the NSLD group compared to this group’s peers. Behavioural manifestations are more related to low self-esteem and a greater awareness of one’s problems in the NSLD group [20,21,39,40,41]. On the other hand, adolescents in the NSLD group with behavioural problems have a more adaptive, generalised pattern than their peers since there is no significant relationship between the externalising variables and personal resources, such as self-esteem, socialisation, or awareness of one’s problems. 

Secondly, we expected adolescents with SLD to have greater problems of both types and fewer personal resources than their peers. This study found significant differences between the groups in relation to all of the study variables. The SLD group had significantly higher scores than the NSLD group in internalising and externalising emotional deficits. On the other hand, the adolescents in the NSLD Group had higher scores than the SLD group in terms of personal resources, except regarding AWE. That is, the SLD group scored significantly higher than the NSLD group in anxiety and depression, aggressive behaviours, anger, and defiant behaviour. In addition, this group obtained lower scores in self-esteem and social competence and integration but higher scores in awareness of their problems [19,20,21,22,23,24,25,26]. These results coincide with some studies that have indicated that populations of schoolchildren with specific educational support needs, specifically with SLD, tend to have greater levels of internalising [19,26] and externalising [18,23,24] emotional difficulties than their normative peers. In particular, some studies indicate that 70% of adolescents with SLD exhibit anxiety problems [19,42], and more than 20% have behavioural disorders [18,20]. Therefore, it seems that having SLD in adolescence can be a vulnerability factor and trigger for emotional difficulties and can endanger mental health and psychological well-being. Adolescents with SLD show higher levels of anxiety and depression than their peers [11,12,13,18,19,20,21,22,23], as well as a higher percentage of externalising problems, such as aggression, anger, and defiant and antisocial behaviour, as noted in other research [20,24]. Furthermore, other studies have found that students with SLD have lower scores in certain personal resources, such as self-esteem [20,21,22,23]. On the other hand, other research [27,28] indicates that this deficit only manifests itself in specific dimensions of self-esteem, linked to academic competencies. Self-esteem has a strong hierarchical and factorial component that promotes mental health [1,37] by being able to present different assessments in each dimension (academic, social, family, etc.). However, in the case of SLD, this influence seems to be easy to extrapolate from specific dimensions to global self-esteem. Along these lines, adolescents with SLD also appears to have a worse perception of their own social competence and integration. Some studies indicate that these low-level social skills limit expectations of social success and negatively influence psychological well-being [29,39]. There are few studies related to the perception of psychological problems among normative school students and their peers with SLD [5]. In our study, we found that adolescents with SLD were more aware of their own emotional difficulties and their lack of ability to resolve conflicts than their peers, becoming a multiplier source of their emotional maladjustment. Furthermore, repeated frustration regarding experiences of failure or the non-achievement of their own learning or personal goals produces anxiety and depression and impairs the mental health of adolescents [1,2,3,4,5,26,27,28], causing them to develop negative thoughts, low expectations for the future, and negative anticipatory thinking regarding the negative, consequences of their own actions [19,26], which would explain the differences found between the groups. 

In short, adolescents often present internalising (anxiety and depression) and externalising (behavioural disorders, aggressiveness, anger, and defiant behaviour) emotional imbalances, and limited personal resources (low self-esteem, poor social competence and integration, and a high level of awareness of their own emotional difficulties). In particular, schoolchildren with SLD present a more maladaptive pattern in adolescence than their peers without SLD since they have very limited adequate strategies for promoting their psychological well-being [18,21,24,28] and display internalising and externalising symptomatology such as low self-esteem and social competence, becoming a self-perceived stressor that damages their self-image [20,23,29]. In addition, they have poorer social skills and exhibit problems regarding social competence and integration, increasing their emotional difficulties and, sometimes, turning into situations of social isolation, which, in turn, influences emotional deficits [11,12,13]. The attributional system of adolescents with SLD detects their own externalising emotional difficulties, establishing as a cause their own personal and social limitations in relation to following rules [21,26], a behaviour that is not exhibited by their peers. Some research indicates that cognitive deficits of students with neurodevelopmental disorders, especially in their executive function, might influence both poor academic outcomes and attributional and emotional problems [22,27,40]. Therefore, the emotional adjustment problems experienced by adolescents with and without SLD negatively affect their psychological well-being and their personal and social functioning.

## 5. Conclusions

The findings of this study highlight the emotional challenges faced by adolescents with specific learning difficulties and their peers as well as the significant risks they pose to their psychological well-being. The internalizing and externalizing symptomatology, coupled with limited personal resources, particularly in terms of self-esteem and socialisation, make students with SLD a highly vulnerable group and increase their risk of mental health issues. It is evident that their persistent experience of academic failure throughout their academic career serves as a breeding ground for the development of various mental health problems.

These results should be taken with caution, as research indicates that boys are more likely to show externalising behaviours while girls generally show more internal emotional deficits [5,41]. In our study, the sample of adolescents with SLD and without SLD mostly consisted of boys, which implies the need to replicate the study in larger and gender-balanced samples. In future studies, it would be advisable to compare the results obtained with those collected using other instruments of measure directed toward other informants, such as peers, teachers, or parents, especially with respect to internalising problems, given the possible influence of subjectivity. Causal analyses could also be performed to establish the relationship between adolescents with and without SLD and emotional imbalances, using regression methodologies or longitudinal studies early on at the start of compulsory schooling.

The results of this study have relevant educational implications since they highlight the need for prevention in the educational stages before adolescence in order to promote personal resources and psychological well-being. The findings underscore the need to develop and implement educational programmes of psychological intervention in schools to reduce the emotional distress presented by adolescents, with content that addresses emotional deficits from the perspective of general mental health [3,4,6]. In addition, teachers must be encouraged to develop an adequate perception of adolescents with SLD and without them, in order to avoid categorisations based on their low learning abilities or maladaptive attributions in relation to their motivation and promote a positive view of their strengths and learning possibilities [1,13,22]. In the case of SLD, psychoeducational interventions could be centred on improving cognitive language skills and reattributing their successes and failures.

## Figures and Tables

**Table 1:** Description of the scales and the reliability of the instrument.

	Scales	*n*	*S* min–max	*α_t_*	*α_e_*
Internalising problems (INTER)	1. ANX	10	10–50	0.87	0.87
	2. DEP	14	14–70	0.91	0.96
Externalising problems (EXTER)	3. AGR	7	7–35	0.91	0.81
	4. ANG	8	8–40	0.89	0.88
	5. DEF	3	3–15	0.87	0.91
	6. ANT	8	8–40	0.71	0.91
Personal Resources PR	7. SEL	7	7–35	0.93	0.95
	8. SOC	9	9–45	0.93	0.87
	9. AWE	7	7–35	0.93	0.88

Note. n (number of items), *S* min–max (minimum and maximum score of the scale), *α_t_* (theoretical reliability test), and *α_e_* (reliability of the test in the study sample). Scales: 1.—ANX (Anxiety), 2.—DEP (Depression), 3.—AGR (Aggression), 4.—ANG (Anger Control), 5.—DEF (Defiant Behaviour), 6.—ANT (Antisocial Behaviour), 7.—SEL (Self-Esteem), 8.—SOC (Social Competence and Integration), and 9.—AWE (Awareness of problems).

**Table 2:** Correlations between variables (upper matrix—SLD group and lower matrix—NSLD group).

	1	2	3	4	5	6	7	8	9
1. ANX		0.82 **	0.52 **	0.66 **	0.70 **	0.45 **	−0.79 **	−0.51 **	0.77 **
2. DEP	0.71 **		0.66 **	0.79 **	0.74 **	0.60 **	−0.78 **	−0.46 **	0.77 **
3. AGR	0.20	0.35 *		0.83 **	0.67 **	0.72 **	−0.51 **	−0.21	0.71 **
4. ANG	0.56 **	0.51 **	0.43 **		0.82 **	0.75 **	−0.63 **	−0.25	0.75 **
5. DEF	0.30	0.30	0.61 **	0.34 *		0.72 **	−0.63 **	−0.024	0.64 **
6. ANT	0.16	0.20	0.72 **	0.42 **	0.59 **		−0.56 **	−0.35 *	0.60 **
7. SEL	−0.58 **	−0.61 **	−0.36 *	−0.25	−0.52 **	−0.30		0.68 **	−0.78 **
8. SOC	−0.40 **	−0.56 **	−0.14	−0.26	−0.26	−0.50	0.62 **		−0.44 **
9. AWE	0.53 **	0.66 **	0.24	0.39 *	0.23	0.05	−0.33 *	−0.30	

Note: *: *p* < 0.05, **: *p* < 0.001. 1. ANX (Anxiety), 2. DEP (Depression), 3. AGR (Aggression), 4. ANG (Anger Control), 5. DEF (Defiant Behaviour), 6. ANT (Antisocial Behaviour), 7. SEL (Self-Esteem), 8. SOC (Social Competence and Integration), and 9. AWE (Awareness of problems).

**Table 3:** Descriptive statistics of study variables and differences among groups.

Variables	*M (SD)* SLD	*M (SD)* NSLD	*t*	*d.f.*	*p*	*d*
ANX	2.53 (0.88)	1.93 (0.65)	3.41	71.9	0.001	0.77
DEP	2.23 (1.09)	1.49 (0.64)	3.67	62.9	0.001	0.56
AGR	1.57 (0.58)	1.26 (0.33)	2.60	62.2	0.012	0.57
ANG	2.33 (0.91)	1.76 (0.67)	3.17	71.7	0.002	0.71
DEF	1.73 (0.95)	1.29 (0.35)	2.75	49.7	0.008	0.84
ANT	1.40 (0.69)	1.15 (0.25)	2.06	49.1	0.045	0.48
SEL	3.38(1.28)	4.22 (0.71)	−3.62	61.2	0.001	0.81
SOC	3.52 (0.92)	4.09 (0.55)	−3.34	64.2	0.001	0.74
AWE	2.57 (1.01)	2.04 (0.73)	2.65	71.2	0.010	0.59

Note: *M* (Mean) *SD* (Standard deviation), *t* (t of Student Welch adjustment) *d.f.* (degrees of freedom) SLD (specific learning disabilities), and NSLD (no specific learning disabilities). ANX (Anxiety), DEP (Depression), AGR (Aggression), ANG (Anger Control), DEF (Defiant Behaviour), ANT (Antisocial Behaviour), SEL (Self-Esteem), SOC (Social Competence and Integration), and AWE (Awareness of problems).

## Data Availability

The data presented in this study are available on request from the corresponding author. The data are not publicly available due to it is embargoed for a period of five years, property of the research group.
